# Differential Roles of Key Brain Regions: Ventral Tegmental Area, Locus Coeruleus, Dorsal Raphe, Nucleus Accumbens, Caudate Nucleus, and Prefrontal Cortex in Regulating Response to Methylphenidate: Insights from Neuronal and Behavioral Studies in Freely Behaving Rats

**DOI:** 10.3390/ijms25115938

**Published:** 2024-05-29

**Authors:** Nachum Dafny, Catherine Claussen, Emilee Frazier, Yin Liu

**Affiliations:** Department of Neurobiology and Anatomy, McGovern Medical School, University of Texas Health Science Center, 6431 Fannin Street, Houston, TX 77030, USA; claussen.catrin.n@uth.tmc.edu (C.C.); yin.liu@uth.tmc.edu (Y.L.)

**Keywords:** methylphenidate, behavior, neuronal recording, VTA, LC, DR, NAc, CN, PFC

## Abstract

A total of 3102 neurons were recorded before and following acute and chronic methylphenidate (MPD) administration. Acute MPD exposure elicits mainly increases in neuronal and behavioral activity in dose–response characteristics. The response to chronic MPD exposure, as compared to acute 0.6, 2.5, or 10.0 mg/kg MPD administration, elicits electrophysiological and behavioral sensitization in some animals and electrophysiological and behavioral tolerance in others when the neuronal recording evaluations were performed based on the animals’ behavioral responses, or amount of locomotor activity, to chronic MPD exposure. The majority of neurons recorded from those expressing behavioral sensitization responded to chronic MPD with further increases in firing rate as compared to the initial MPD responses. The majority of neurons recorded from animals expressing behavioral tolerance responded to chronic MPD with decreases in their firing rate as compared to the initial MPD exposures. Each of the six brain areas studied—the ventral tegmental area, locus coeruleus, dorsal raphe, nucleus accumbens, prefrontal cortex, and caudate nucleus (VTA, LC, DR, NAc, PFC, and CN)—responds significantly (*p* < 0.001) differently to MPD, suggesting that each one of the above brain areas exhibits different roles in the response to MPD. Moreover, this study demonstrates that it is essential to evaluate neuronal activity responses to psychostimulants based on the animals’ behavioral responses to acute and chronic effects of the drug from several brain areas simultaneously to obtain accurate information on each area’s role in response to the drug.

## 1. Introduction

Methylphenidate (MPD), also known as Ritalin, is a psychostimulant approved by the Food and Drug Administration (FDA) for the treatment of behavioral disorders such as Attention Deficit Hyperactive Disorder (ADHD) and is classified as a Schedule II medication by the United States Drug Enforcement Administration because of its high potential for abuse and dependency [1,2]. The use of MPD has recently increased by many “ordinary” individuals unaffected by ADHD, including college students, young adults, and even the elderly, for cognitive enhancement in demanding jobs, fields, and today’s competitive society, as well as for recreational use [3,4,5,6,7]. Amaral et al. [8] reviewed 224 articles dealing with the use of MPD for cognitive enhancement by the average “ordinary” medical student without a doctor’s prescription. The most frequent justification for the unregulated use of MPD was to improve academic performance. Thus, it is essential to study the properties of acute and repetitive (chronic) use of MPD on “ordinary” subjects. This is a particular concern as MPD abuse is extremely dangerous, with intravenous or intranasal consumption having higher mortality rates than other drugs of abuse, such as cocaine and amphetamines [4,9].

MPD consumption results in biochemical, molecular, and neuronal plasticity in the central nervous system (CNS) as a result of inhibiting the reuptake of serotonin (5HT), norepinephrine (NE), and dopamine (DA) from the synaptic cleft back to presynaptic terminals, thereby prolonging and increasing the amount of these neurotransmitters available in the post-synaptic cleft [4,5,9,10,11,12,13,14]. The resulting areas of the brain with increased 5HT, NE, and DA available within the post-synaptic cleft have been shown to be associated with addiction and reward [4,15,16,17,18,19,20,21].

The main sources of 5HT, NE, and DA to the CNS reward circuit are from the dorsal raphe (DR), locus coeruleus (LC), and ventral tegmental area (VTA), respectively [22]. These nuclei ascend and descend to the nucleus accumbens (NAc), caudate nucleus (CN), prefrontal cortex (PFC), and other brain areas within the motive or reward neuronal centers [18,23,24,25,26].

Several studies investigating the property of MPD on a single CNS structure of acute and chronic MPD on DR [27], on LC [28], on VTA [14,29], on NAc [14], on CN, and on the PFC [20] reported that MPD exerts similar effects on each of the above six brain areas. Based on our preliminary study using simultaneous neuronal recordings from the above six brain areas, we hypothesize that each of the six brain areas plays a different role in MPD action. To prove this, the behavioral and neuronal activities from the respective brain areas were recorded simultaneously, and it was observed, as hypothesized, that the respective response to MPD from each brain area (DR, LC, VTA, NAc, CN, and PFC) was, in fact, different.

In previous studies using recordings from one brain area using dose–response protocols of acute and chronic 0.6, 2.5, and 10.0 mg/kg MPD [27,28], it was observed that the same dose of 0.6, 2.5, or 10.0 mg/kg MPD elicited behavioral sensitization in some animals and behavioral tolerance in others. It can, therefore, be assumed that the neuronal activity recordings from each of the above six brain areas in animals expressing behavioral sensitization will be significantly different from the neuronal recordings of animals expressing behavioral tolerance to chronic MPD exposure as compared to the effect of the initial MPD dose.

Therefore, the objective of this study is to test the following hypothesis: (1) The chronic effect of 0.6, 2.5, or 10.0 mg/kg MPD will elicit in some animals behavioral and neuronal sensitization, while in others using the same MPD dose, it will elicit behavioral and neurophysiological tolerance as compared to the initial effect of the drug, respectively, for each dose. (2) The neuronal activity from each of the six proposed brain areas recorded from animals with behavioral sensitization to chronic MPD exposure will exhibit mainly excitation to MPD treatment, while the neuronal activities recorded from those with behavioral tolerance will exhibit mainly attenuation in their neuronal activity firing rate in response to chronic MPD treatment as compared to the acute (initial) MPD effects. (3) Each of the above six brain areas will respond differently to 0.6, 2.5, or 10.0 mg/kg MPD, such that the ratio of the number of neuronal units that respond to one of the above MPD doses by excitation to attenuation will be significantly different among the respective brain areas, indicating each brain area plays a different role in the response to MPD.

## 2. Results

### 2.1. Locomotor Behavioral Expression

A total of 158 adult male SD rats were evaluated—12, 13, 45, 41, and 47 were used following time (control), saline, 0.6, 2.5, and 10.0 mg/kg MPD, respectively.

### 2.2. Time and Saline (Sal) Control (Figure 1)

**Figure 1:**
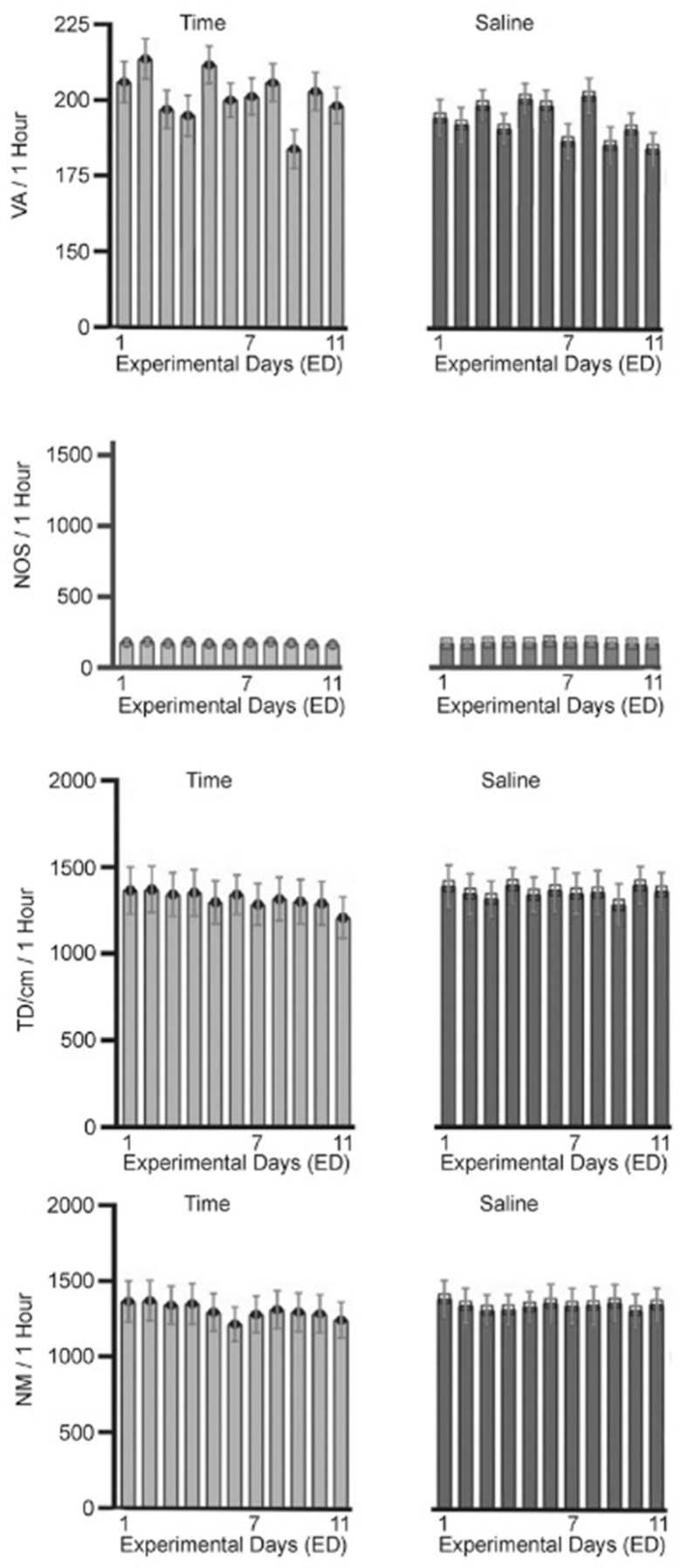
The figure summarizes the four locomotor behavioral expressions of the time (N = 12) and saline (N = 13) control groups for the number of movements (NM), total distance (TD) traveled, number of stereotypic (NOS) activity, and vertical activity (VA) during the 11 experimental recording days (ED) before and after saline injection. Animals in the time control group did not receive any injections. In the saline control group, animals were injected daily with saline from ED 2 to ED 7. The four locomotor behavioral expressions exhibited similar levels of locomotor behavioral activity with no significant fluctuation, indicating that the animal handling and injection procedures during the experimental days did not modify the four locomotor behavioral expressions.

Animals in the time control group over all eleven recording days exhibited similar levels of locomotor activity (Figure 1—TD, NM, NOS, and VA). Comparing the locomotor activity of the time control on ED 1 to the other recording days shows no significant differences (F = 0.0486, *p* < 0.982; one-way RM ANOVA). Comparing the locomotor activities of ED 1 following saline injection to its baseline (BL) recording at ED1 and on the other recording days following saline injections shows no significant differences across the recording days (F = 0.0413, *p* < 0.952; one-way RM ANOVA). This demonstrates that the handling, injection procedure, and environment had no effect on the animals’ locomotor behaviors over all experimental days (Figure 1).

### 2.3. Effect of Acute and Chronic 0.6, 2.5, and 10.0 mg/kg MPD on Number of Movements (NM) (Figure 2 Left Side Histogram—All)

**Figure 2:**
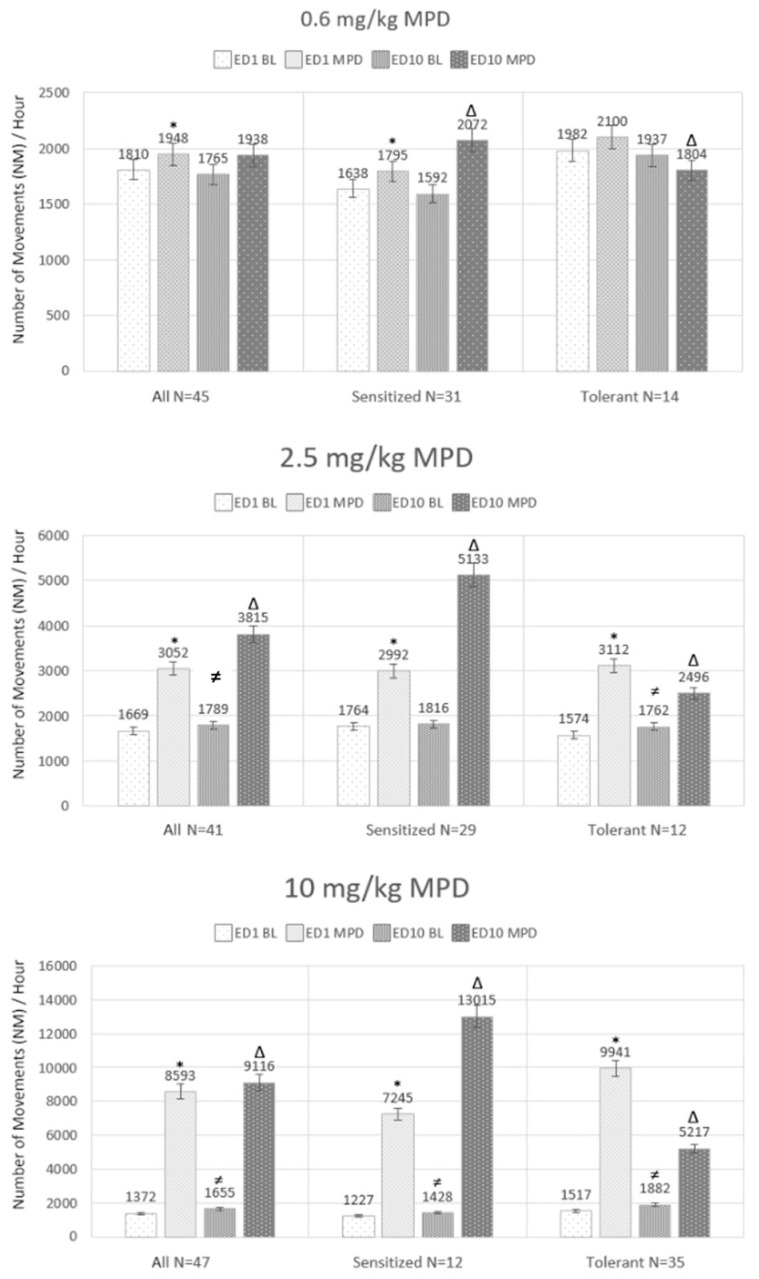
Summarizes the number of movement (NM) counts before and following acute and chronic 0.6, 2.5, and/or 10.0 mg/kg MPD in all the experimental animals (under ALL), as well as only the NM count from animals expressing behavioral sensitization (under Sensitized) or behavioral tolerance (under Tolerance) following chronic MPD as compared to the initial MPD exposure. The first column summarizes the NM baseline of experimental day 1 (ED1 BL). The second is the effect of acute MPD on NM (ED1 MPD). The third column is the NM counts on ED10 BL after six daily MPD treatments and three washout days. The fourth column summarizes the NM count following the MPD rechallenge at ED10. The figure shows that the three animals’ groups (All, Sensitized, and Tolerance) responses are significantly different from the three acute and chronic MPD exposures. ED = experimental recording day; MPD = methylphenidate; * = indicate significant (*p* < 0.05) different from ED1 MPD/ED1 BL; ≠ indicate significant (*p* < 0.05) different from ED10 BL/ED1 BL; Δ = indicate significant (*p* < 0.05) different from ED10 MPD/ED1 MPD.

Figure 2 summarizes the acute and chronic effects of 0.6, 2.5, and 10.0 mg/kg MPD on NM in all three groups (all, sensitized, and tolerant).

#### 2.3.1. Acute Effect of MPD on All Groups (ED1 MPD/ED1 BL; Figure 2 Left Side Histogram)

Acute 0.6, 2.5, and 10.0 mg/kg MPD elicit significant (F = 28.034, *p* < 0.01; F = 32.031, *p* < 0.001; F = 43.734, *p* < 0.001) differences compared to ED1 BL. This is demonstrated by the respective increases in the NM (Figure 2: All ED1 MPD/ED1 BL), using the RM ANOVA.

#### 2.3.2. Baseline Effects after Chronic MPD of All Groups (ED10 BL/ED1 BL; Figure 2: Left Side)

The ED10 BL activities after six days of repetitive (chronic) 0.6, 2.5, or 10.0 mg/kg MPD and three washout days exhibit no change, increase, or increase in baseline NM, respectively (F = 0.152, *p* < 0.713; F = 20.016, *p* < 0.05; F = 24.405, *p* < 0.001; one-way RM ANOVA).

#### 2.3.3. Chronic Effect of MPD on All Groups (ED10 MPD/ED1 MPD; Figure 2: Left Side)

The chronic effects of 0.6, 2.5, and 10.0 mg/kg compared to the initial effects of MPD at ED1 MPD (ED10 MPD/ED1 MPD) resulted in similar effects as the acute 0.6 mg/kg MPD animals’ group, with further significant (F = 19.842, *p* < 0.01; F = 23.704. *p* < 0.001) increases in the 2.5 and 10.0 mg/kg MPD groups as compared to the initial MPD effects. This further significant increase indicates that behavioral sensitization was expressed.

### 2.4. Effect of Acute and Chronic 0.6, 2.5, and 10.0 mg/kg MPD on NM on Behaviorally Sensitized Groups (ED1 MPD/ED1 BL; Figure 2: Middle Histograms—Sensitized)

The NM of the behaviorally sensitized group to acute 0.6, 2.5, or 10.0 mg/kg MPD elicits significant differences (F = 24.044, *p* < 0.05; F = 29.313, *p* < 0.001; F = 32.425, *p* < 0.001) compared to ED1 BL, as indicated by the increase in the respective NM using the RM ANOVA as compared to ED1 BL activity, respectively.

### 2.5. Baseline Effects after Chronic MPD on NM on Behavioral Sensitized Groups (ED10 BL/ED1 BL; Figure 2: Middle Histograms)

The ED10 BL activities after six days of repetitive (chronic) 0.6 or 2.5 mg/kg MPD were about the same as ED1 BL (F = 0.178, *p* < 0.872; F = 0.916, *p* < 0.705); while the 10.0 mg/kg group exhibited a significant increase in ED10 BL/ED1 BL (Figure 2 sensitized; F = 48.485, *p* < 0.001; one-way RM ANOVA).

### 2.6. Effect of Chronic MPD on the Sensitized Groups (ED10 MPD/ED1 MPD; Figure 2: Middle Histograms—Sensitized)

The chronic effects of 0.6, 2.5, or 10.0 mg/kg compared to the initial effects of MPD at ED1 MPD (ED10 MPD/ED1 MPD) resulted in significant increases as compared to the acute effects of the drug on ED1 (F = 33.610, *p* < 0.001; F = 42.357, *p* < 0.001; F = 59.705, *p* < 0.001). This further significant increase indicates that behavioral sensitization was expressed.

### 2.7. Acute Effect of MPD on Behaviorally Tolerant Groups (ED1 MPD/ED1 BL; Figure 2: Right Histograms)

The behaviorally tolerant animal groups responded to acute 0.6 mg/kg MPD with no significant increase or decrease (F = 0.204, *p* < 0.707) and significant increases to acute 2.5 and 10.0 mg/kg MPD (F = 41.581, *p* < 0.001; F = 52.548, *p* < 0.001).

### 2.8. Baseline Effects after Chronic MPD on NM of Behaviorally Tolerant Groups (ED10 BL/ED1 BL; Figure 2: Right Histograms—Tolerance)

The ED10 BL activities after six days of repetitive (chronic) 0.6, 2.5, or 10.0 mg/kg MPD and three washout days were roughly the same when compared to ED1 BL in the 0.6 mg/kg MPD (F = 0.152, *p* < 0.8160) and significant increases (F = 28.426, *p* < 0.001; and F = 33.375, *p* < 0.001) in the groups that were treated with 2.5 and 10.0 mg/kg MPD, respectively, (one way RM ANOVA) in ED10 BL/ED1 BL.

### 2.9. Chronic Effect of MPD on Behaviorally Tolerant Groups (ED10 MPD/ED1 MPD; Figure 2: Right Histograms)

The rechallenge (chronic) effects of 0.6, 2.5, or 10.0 mg/kg after six daily MPD exposures and three washout days compared to the initial effects of MPD at ED1 MPD (ED10 MPD/ED1 MPD) resulted in a significant reduction in locomotor activity behaviors compared to the initial effects of each MPD dosage. This significant decrease, as compared to the acute effect of the drug on ED1, indicates that behavioral tolerance was expressed following chronic 0.6, 2.5, and 10.0 mg/kg MPD, respectively (F = 29.671, *p* < 0.001; F = 33.455, *p* < 0.001; F = 44.781, *p* < 0.001).

The Chi-Square test was used to determine that the ratio for a number of animals who expressed behavioral sensitization vs. behavioral tolerance to chronic 0.6, 2.5, and 10.0 mg/kg MPD was significantly different for each respective MPD dosage (*p* < 0.05; 31:14, 29:12, and 12:35).

### 2.10. Electrophysiology

A total of 3102 neuronal units were recorded and evaluated: 345 neuronal units following Sal injections and 2757 following MPD injections.

### 2.11. Neuronal Units Recording following Exposed to Saline (Sal)

The neuronal units recorded after the first Sal injection on ED1 were VTA—26, LC—60, DR—27, NAc—56, CN—68, and PFC—98. The percentage of units recorded from each brain region that demonstrated no change in activity following single or repeated Sal injections on ED1 and ED10 compared to the initial ED1 Sal injection were as follows: VTA—96%, LC—97%, DR—93%, NAc—98%, CN—92%, and PFC—95%. This observation shows that animal handling, volume injection, and the experimental system do not significantly affect the neuronal activities in the above six brain areas.

### 2.12. Neuronal Units Exposed to MPD (Table 1 and Figure 3)

**Figure 3:**
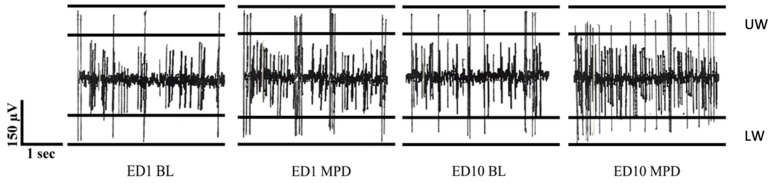
The left first panel is ED1 BL, the second panelis ED1 following 2.5 mg/kg MPD, causing excitation. The third panel is ED10 BL after six daily 2.5 mg/kg MPD and three washout days, showing withdrawal. The last panel is ED10 following 2.5 mg/kg MPD, showing sensitization. All recordings were taken 5 min post-injection.

**Figure 4:**
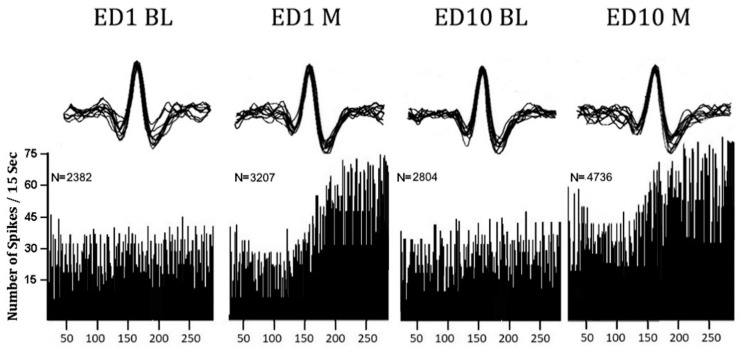
The histogram under ED1 BL represents the recording on ED1 BL after saline injection, and the 2382 numbers are the neuronal activity counts/60 min of ED1 BL. The histogram shows that 2.5 mg/kg MPD (ED1 M) elicits excitation (N = 3207), and the ED10 BL/ED1 BL after six daily 2.5 mg/kg MPD and three washout days (ED10 BL and ED1 BL) exhibit an increase in activity, indicating neurophysiological withdrawal. Rechallenge with 2.5 mg/kg MPD (ED10 M) compared to ED1 MPD shows a significant increase, indicating that neuronal sensitization is expressed.

A total of 2757 neuronal units were recorded and evaluated following single and repeated (chronic) MPD exposures: 927, 922, and 908 following 0.6, 2.5, and 10.0 mg/kg MPD, respectively. Table 1, Table 2 and Table 3 summarize how the neuronal units recorded from the VTA, LC, DR, NAc, CN, and PFC responded to 0.6, 2.5, or 10.0 mg/kg MPD, respectively. Figure 3 shows a representative of typical analog activity on ED1 BL in A, ED1 MPD in B, ED10 BL in C, and ED10 MPD in D, while Figure 4 shows a representative frequency histogram of 60 min PFC unit recordings at ED1 BL, ED1 MPD, ED10 BL, and ED10 MPD. Above each histogram are the sorted 20 superimposed spike patterns and amplitudes that the program counted to produce each of the frequency histograms below.

**Table 1:** Summarizes the effects of acute MPD administration on experimental recording day 1 (ED 1) for 0.6 mg/kg compared to ED1 baseline (ED1 MPD/ED1 BL; left side of the table), ED 10BL/ED1 BL neuronal activities after six daily MPD exposures and three washout days in the middle table, and ED10 MPD/ED1 MPD (right side of the table) on the ventral tegmental area (VTA), locus coeruleus (LC), dorsal raphe (DR), nucleus accumbens (NAc), prefrontal cortex (PFC), and caudate nucleus (CN) neuronal activity, respectively. Where N = number of neuronal units in each brain region; ↑ column = number of neuronal units in each brain region that exhibited significant (*p* < 0.05) increases in neuronal activity after acute 0.6 mg/kg MPD exposure; **↓** column = number of neuronal units in each brain region that exhibited significant (*p* < 0.05) decreases in neuronal activity after acute exposure; **%** column = number of neuronal units that exhibited no significant change to MPD exposure. Section A (upper table) summarizes the neuronal activity responses to the 0.6 mg/kg MPD doses in all rats. Section B (middle table) summarizes only the neuronal responses to 0.6 mg/kg MPD from animals exhibiting behavioral sensitization. Section C (lower table) summarizes only the neuronal responses to 0.6 mg/kg MPD recoded from animals exhibiting behavioral tolerance.

					ALL						
			ED1 MPD/ED1 BL Acute Effect			ED10 BL/ED1 BL Withdrawal			ED10 MPD/ED1 MPD Chronic		
**A**	**Dose**	N	↑	↓	•/•	↑	↓	•/•	↑	↓	•/•
**VTA**	**0.6 mg**	171	47	65	59	53	83	35	67	73	31
**LC**		128	29	21	78	47	67	14	43	63	22
**DR**		122	39	19	64	51	57	14	48	58	16
**NAC**		142	41	67	34	15	6	121	88	31	23
**PFC**		145	41	24	80	24	43	78	29	22	94
**CN**		219	92	106	21	65	153	1	103	116	0
	**Total**	927									
					**Sensitized**						
**B**	**Dose**	N	↑	↓	•/•	↑	↓	•/•	↑	↓	•/•
**VTA**	**0.6 mg**	29	2	23	4	8	15	6	13	15	1
**LC**		56	18	10	29	29	23	4	34	18	4
**DR**		27	5	6	16	11	9	7	10	5	12
**NAC**		49	3	42	4	3	1	45	35	6	8
**PFC**		61	18	19	24	17	31	13	27	11	23
**CN**		60	40	20	0	24	35	1	43	16	1
	**Total**	282									
					**Tolerance**						
**C**	**Dose**	N	↑	↓	•/•	↑	↓	•/•	↑	↓	•/•
**VTA**	**0.6 mg**	142	61	42	39	55	77	10	56	72	14
**LC**		72	20	15	37	20	44	8	24	47	1
**DR**		95	33	13	49	38	49	8	39	54	2
**NAC**		93	39	32	22	10	32	51	50	39	4
**PFC**		84	23	5	56	7	12	65	2	11	71
**CN**		159	75	31	53	35	124	0	42	115	2
	**Total**	645									

ED—Experimental Day/BL—Baseline/↑—Significant (*p* < 0.05) Increase/↓—Significant (*p* < 0.05) Decrease/•/•—No Change.

**Table 2:** Summarizes the effects of acute MPD administration on experimental recording day 1 (ED 1) for 2.5 mg/kg compared to ED1 baseline (ED1 MPD/ED1 BL; left side of the table), ED 10BL/ED1 BL neuronal activities after six daily MPD exposures and three washout days in the middle table, and ED10 MPD/ED1 MPD (right side of the table) on the ventral tegmental area (VTA), locus coeruleus (LC), dorsal raphe (DR), nucleus accumbens (NAc), prefrontal cortex (PFC), and caudate nucleus (CN) neuronal activity, respectively. Where N = number of neuronal units in each brain region; ↑ column = number of neuronal units in each brain region that exhibited significant (*p* < 0.05) increases in activity after acute 2.5 mg/kg MPD exposure; **↓** column = number of neuronal units in each brain region that exhibited significant (*p* < 0.05) decreases in activity after acute exposure; **%** column = number of neuronal units that exhibited no significant change to MPD exposure. Section A (upper table) summarizes the neuronal activity responses to the 2.5 mg/kg MPD doses in all rats. Section B (middle table) summarizes only the neuronal responses to 2.5 mg/kg MPD from animals exhibiting behavioral sensitization. Section C (lower table) summarizes only the neuronal responses to 2.5 mg/kg MPD in animals exhibiting behavioral tolerance.

					ALL						
			ED1 MPD/ED1 BL Acute Effect			ED10 BL/ED1 BL Withdrawal			ED10 MPD/ED1 MPD Chronic		
**A**	**Dose**	N	↑	↓	•/•	↑	↓	•/•	↑	↓	•/•
**VTA**	**2.5 mg**	102	68	22	12	45	49	8	52	48	2
**LC**		136	81	25	30	51	81	4	54	71	11
**DR**		94	49	5	40	32	32	27	45	29	20
**NAC**		156	61	72	23	31	15	110	90	51	15
**PFC**		185	81	29	75	66	87	32	71	39	75
**CN**		249	152	77	20	113	125	1	137	112	0
	**Total**	922			**Sensitized**						
**B**	**Dose**	N	↑	↓	•/•	↑	↓	•/•	↑	↓	•/•
**VTA**	**2.5 mg**	40	18	14	8	10	18	2	16	22	2
**LC**		57	37	11	9	39	16	2	41	14	2
**DR**		42	17	1	24	13	22	7	20	17	5
**NAC**		78	16	50	12	7	4	67	44	28	6
**PFC**		115	52	22	41	43	59	13	69	17	29
**CN**		105	47	42	16	69	36	0	79	20	6
	**Total**	437									
					**Tolerance**						
**C**	**Dose**	N	↑	↓	•/•	↑	↓	•/•	↑	↓	•/•
**VTA**	**2.5 mg**	62	52	5	5	44	15	3	27	35	0
**LC**		79	58	16	5	28	47	4	11	59	9
**DR**		52	33	4	15	21	11	20	23	14	15
**NAC**		78	42	17	19	5	11	62	41	20	17
**PFC**		70	29	7	34	23	28	19	2	22	46
**CN**		144	127	5	12	19	125	0	115	28	1
	**Total**	485									

ED—Experimental Day/BL—Baseline/↑—Significant (*p* < 0.05) Increase/↓—Significant (*p* < 0.05) Decrease/•/•—No Change.

**Table 3:** Summarizes the effects of acute MPD administration on experimental recording day 1 (ED 1) for 10.0 mg/kg compared to ED1 baseline (ED1 MPD/ED1 BL; left side of the table), ED 10BL/ED1 BL neuronal activities after six daily MPD exposures and three washout days (middle of the table), and ED10 MPD/ED1 MPD (right side of the table) on the ventral tegmental area (VTA), locus coeruleus (LC), dorsal raphe (DR), nucleus accumbens (NAc), prefrontal cortex (PFC), and caudate nucleus (CN) neuronal activity, respectively. Where N = number of neuronal units in each group; ↑ column = number of neuronal units in each brain region that exhibited significant (*p* < 0.05) increases in activity after acute 10.0 mg/kg MPD exposure; ↓ column = number of neuronal units in each brain region that exhibited significant (*p* < 0.05) decreases in activity after acute exposure; % column = number of neuronal units that exhibited no significant change to MPD exposure. Section A (upper table) summarizes the neuronal activity responses to the 10.0 mg/kg MPD doses in all rats. Section B (middle table) summarizes only the neuronal responses to 10.0 mg/kg MPD from animals exhibiting behavioral sensitization. Section C (lower table) summarizes only the neuronal responses to 10.0 mg/kg MPD in animals exhibiting behavioral tolerance.

					ALL						
			ED1 MPD/ED1 BL Acute Effect			ED10 BL/ED1 BL Withdrawal			ED10 MPD/ED1 MPD Chronic		
**A**	**Dose**	N	↑	↓	•/•	↑	↓	•/•	↑	↓	•/•
**VTA**	**10.0 mg**	99	59	21	19	23	76	0	27	92	0
**LC**		141	93	35	13	64	77	0	53	85	3
**DR**		113	72	11	30	37	61	15	39	61	13
**NAC**		133	76	53	4	22	13	98	67	59	7
**PFC**		159	122	17	20	107	31	21	99	17	43
**CN**		263	187	62	14	61	202	0	133	130	0
	**Total**	908									
					**Sensitized**						
**B**	**Dose**	N	↑	↓	•/•	↑	↓	•/•	↑	↓	•/•
**VTA**	**10.0 mg**	42	21	18	3	15	27	0	16	26	0
**LC**		35	19	8	8	18	16	1	31	3	1
**DR**		20	15	0	5	4	15	1	3	16	1
**NAC**		57	33	24	0	11	4	42	40	16	1
**PFC**		116	87	14	15	86	25	5	98	9	9
**CN**		40	38	2	0	31	9	0	29	10	1
	**Total**	310									
					**Tolerance**						
**C**	**Dose**	N	↑	↓	•/•	↑	↓	•/•	↑	↓	•/•
**VTA**	**10.0 mg**	57	38	3	16	11	46	0	9	48	0
**LC**		106	67	22	17	31	69	6	10	91	5
**DR**		93	59	10	24	32	46	15	36	45	13
**NAC**		76	40	34	2	1	4	71	28	41	7
**PFC**		43	35	3	5	21	6	16	1	8	34
**CN**		223	138	58	27	33	184	6	184	38	1
	**Total**	598									

D—Experimental Day/BL—Baseline/↑—Significant (*p* < 0.05) Increase/↓—Significant (*p* < 0.05) Decrease/•/•—No Change.

### 2.13. Acute Response to 0.6 mg/kg MPD in Animals Expressing Behavioral Sensitization (ED1 MPD/ED1 BL; Table 1B and Figure 5A)

Table 1B summarizes the neuronal responses following acute and chronic 0.6 mg/kg MPD exposure. A comparison was made between the proportion of neuronal units that responded to acute MPD in the six brain regions of animals expressing behavioral sensitization and chronic 0.6 mg/kg MPD. It was found that the proportions of responding neuronal units are significantly different among the six brain regions (χ^2^ (5, 282) = 67.66; *p* < 0.0001). The data were then fit into a logistic regression model, and a post hoc comparison was performed. The proportion of neuronal units responding to MPD acutely in region CN (98%) is significantly higher compared to the averaged response rates of all other regions (adjusted *p*-value = 0.01), while the proportion of responding neuronal units in region DR (41%) is significantly lower than the average of all other regions (*p* < 0.0001).

**Figure 5:**
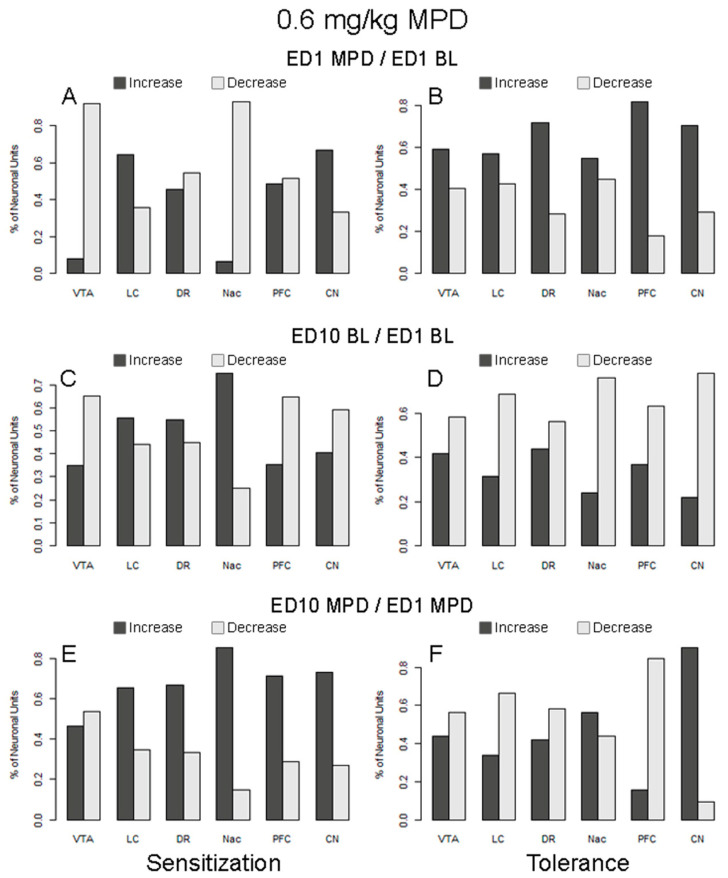
The figure summarizes the percentage of neuronal units from six brain regions responding to 0.6 mg/kg MPD with significantly increased or decreased firing rates. The figures on the left-hand side (**A**,**C**,**E**) summarize responses from animals expressing behavioral sensitization, and the right-hand side (**B**,**D**,**F**) summarize responses from animals expressing behavioral tolerance. The figures in the top row (**A**,**B**) represent the responses following acute MPD (ED1 MPD/ED1 BL), the middle row (**C**,**D**) summarizes the changes to the baseline responses (ED10 BL/ED1 BL), and the bottom row (**E**,**F**) summarizes responses following chronic (rechallenge) MPD (ED10 MPD/ED1 MPD). The columns above the following areas describe the recording obtained: VTA—ventral tegmental area; LC—locus coeruleus; DR—dorsal raphe; NAc—nucleus accumbens; PFC—prefrontal cortex; CN—caudate nucleus.

A comparison was also made between the ratio of neuronal units responding to MPD with increased vs. decreased firing rates among the six different brain regions to 0.6 mg/kg MPD rechallenged at ED10 (Figure 5A). It was found that the ratios are significantly different among these regions (χ^2^ (5, 206) = 56.44; *p* < 0.0001). Further post hoc comparison indicates that the ratio of neuronal units that respond to MPD with increased vs. decreased firing rates in the region CN is significantly higher (adjusted *p*-value = 0.0001), while the ratios in regions NAc and VTA are significantly lower than the average of all other regions, with adjusted *p*-values of 0.001 and 0.03, respectively.

### 2.14. Acute Response to 0.6 mg/kg MPD in Animals Expressing Behavioral Tolerance (ED1 MPD/ED1 BL; Table 1C and Figure 5B)

Table 1C summarizes the neuronal responses following acute and chronic MPD exposure recorded from animals expressing behavioral tolerance to chronic MPD when compared to the initial (acute) response to MPD. The neuronal recordings from animals expressing behavioral tolerance to chronic MPD exhibited different proportions of neuronal units that respond among the six brain regions (χ^2^ (5, 645) = 56.80; *p* < 0.0001). The proportion of neuronal units responding to MPD acutely in region NAc (76%) is significantly higher (*p* = 0.0008), while the proportion of responding neuronal units in region PFC (33%) is significantly lower than the average of all other regions (adjusted *p*-values < 0.0001).

In addition, it was found that the ratios of neuronal units responding to MPD with increased vs. decreased firing rates are only marginally different among these regions (Figure 5B; χ^2^ (5, 389) = 11.59; *p* = 0.04). In all six regions, there is a higher percentage of neuronal units that respond to MPD with increased firing rates than those with decreased firing rates.

### 2.15. ED10 BL Neuronal Activity of Animals Expressing Behavioral Sensitization to Chronic 0.6 mg/kg MPD Compared to ED1 BL (ED10 BL/ED1 BL; Table 1B and Figure 5C)

The proportions of neuronal units that changed their ED10 BL after six daily 0.6 mg/kg MPD and three washout days are significantly different among six brain regions (χ^2^ (5, 282) = 137.01; *p* < 0.0001). The proportion of neuronal units that modified their ED10 BL is significantly higher in region CN (98%) when compared to the average firing rates of all other regions (adjusted *p*-values 0.008), while the modified rate in region NAc (8%) is significantly lower than the average of all other regions (*p* < 0.0001).

A comparison between the respective ratios of neuronal units responding to MPD with increased vs. decreased firing rates among the six different brain regions was made (Figure 5C). It was found that the ratios are not significantly different among these regions (χ^2^ (5, 206) = 7.90; *p* = 0.162). Although the ratio of neuronal units with increased vs. decreased firing rates in the region of NAc was observed to be high, statistical significance was not achieved due to the small sample size (only four neuronal units were recorded in this region).

### 2.16. ED10 BL Neuronal Activity of Animals Expressing Behavioral Tolerance to Chronic 0.6 mg/kg MPD Compared to ED1 BL (ED10 BL/ED1 BL; Table 1C and Figure 5D)

The proportions of neuronal units that changed their ED10 BL/ED1 BL are significantly different among the six brain regions (χ^2^ (5, 646) = 283.61; *p* < 0.0001). The proportion of neuronal units where ED10 BL changed in the CN (99%) is significantly higher than the average of all other regions (*p* = 0.0006). The proportions of ED10 BL neuronal units’ activity that are significantly lower as compared to ED1 BL were observed in NAc (45%) and PFC (30%) (adjusted *p*-values < 0.0001).

In addition, a significant difference was observed in the ratio of neuronal units responding to MPD with increased vs. decreased firing rates following 0.6 mg/kg MPD among the six regions (Figure 5D; χ^2^ (5, 503) = 19.52; *p* = 0.002). Further post hoc comparison indicates that the ratio of neuronal units expressing increased vs. decreased firing rates after six daily 0.6 mg/kg MPD is significantly lower in the CN (adjusted *p* = 0.03) than the average of all other regions.

### 2.17. Neuronal Response to 0.6 mg/kg MPD Rechallenge in Animals Expressing Behavioral Sensitization (ED10 MPD/ED1 MPD; Table 1B and Figure 5E)

The proportions of responding neuronal units to chronic MPD treatment compared to the initial 0.6 mg/kg MPD are significantly different among the six brain regions (χ^2^ (5, 282) = 49.69; *p* < 0.0001). Specifically, the proportions of neuronal units that responded to MPD rechallenge in the DR (56%) and PFC (62%) are significantly lower compared to the average response rates of all other regions (adjusted *p*-values < 0.0001).

A comparison was also made between the ratio of neuronal units that responded to MPD with increased vs. decreased firing rates among the six different brain regions (Figure 5E). It was found that the ratios are significantly different among these regions (χ^2^ (5, 233) = 12.74; *p* = 0.026). Further post hoc comparison indicates that the ratio of neuronal units with increased vs. decreased firing rates is significantly higher in NAc (adjusted *p*-value 0.04) but significantly lower in the VTA (adjusted *p*-value 0.03) when compared to the average of all other regions.

### 2.18. Neuronal Response to 0.6 mg/kg MPD Rechallenge in Animals Expressing Behavioral Tolerance (ED10 MPD/ED1 MPD; Table 1C and Figure 5F)

The proportions of responding neuronal units to chronic MPD treatment are significantly different among the six brain regions (χ^2^ (5, 645) = 385.12; *p* < 0.0001). The proportion of neuronal units that responded to MPD rechallenge is significantly lower in region PFC (15%; adjusted *p*-values < 0.00001).

In addition, it was found that the ratios of neuronal units that responded to MPD with increased vs. decreased firing rates are significantly different among these regions (Figure 5F; χ^2^ (5, 551) = 114.1; *p* < 0.0001). Further post hoc comparison indicates that the ratio of neuronal units with increased vs. decreased firing rates is significantly higher in the region CN (adjusted *p* < 0.0001) but significantly lower in the region PFC (adjusted *p*-value 0.04) than all other regions.

### 2.19. Neuronal Response to Acute and Chronic MPD Exposure from All Animals to Chronic 0.6 mg/kg MPD (Table 1A)

Table 1A summarizes the effects of MPD on all the neuronal units recorded following 0.6 mg/kg MPD. The proportion of responding neuronal units to acute and chronic MPD exposure (ED1 MPD/ED1 BL; ED10 MPD/ED1 MPD), as well as the neuronal activities of ED10 BL after six daily 0.6 mg/kg MPD treatments compared to ED1 BL (ED10 BL/ED1 BL), were significantly different among the six brain regions (χ^2^ (5, 307) = 522.21; *p* < 0.0001). Moreover, the neuronal units recorded in response to 0.6 mg/kg MPD from all animals (Table 1A) were significantly different from the units recorded from animals who responded with behavioral sensitization (Table 1B; χ^2^ (10, 756) = 641.82; *p* < 0.0001) and tolerance (Table 1C; χ^2^ (10, 847) = 724.04; *p* < 0.0001). The above observations indicate that it is essential to evaluate the neuronal activities based on the animal’s behavioral response to the chronic effects of the drug.

### 2.20. Acute Response to 2.5 mg/kg MPD in Animals Expressing Behavioral Sensitization (ED1 MPD/ED1 BL; Table 2B and Figure 6A)

The proportion of neuronal units responding to acute treatment of 2.5 mg/kg MPD in six brain regions and chronic MPD exposure in animals expressing behavioral sensitization was compared. It was found that the proportions of responding neuronal units are significantly different among the six brain regions (χ^2^ (5, 437) = 42.20; *p* < 0.0001). The data were then fit into a logistic regression model, and a post hoc comparison was performed. The proportion of neuronal units responding to MPD acutely in region DR (43%) is significantly lower compared to the averaged response rates of all other regions (*p* < 0.0001).

**Figure 6:**
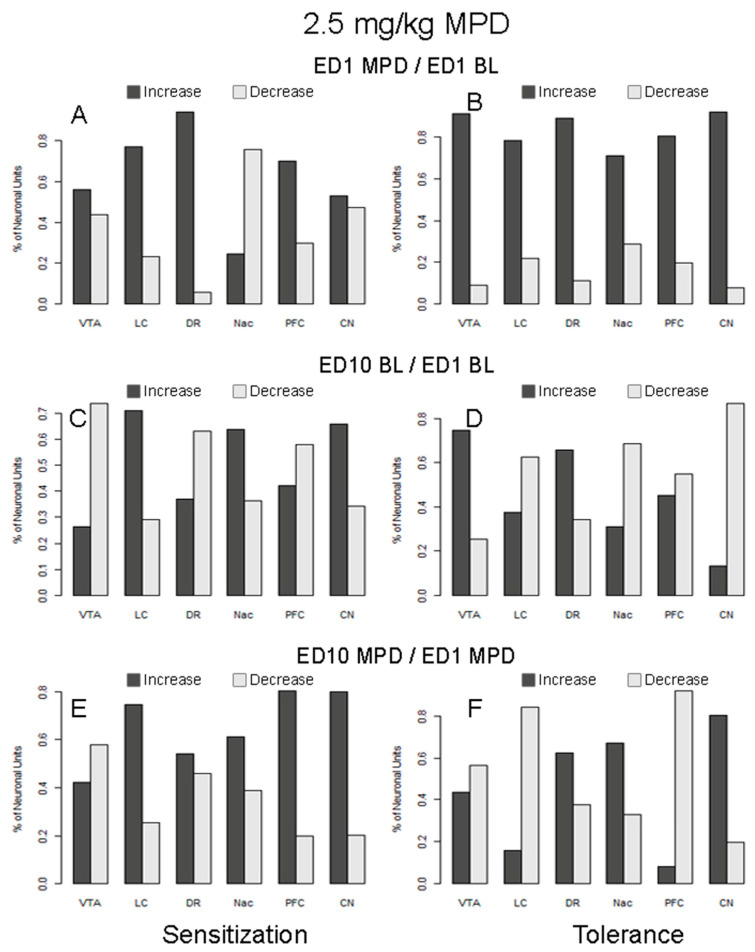
This figure summarizes the percentage of neuronal units from six brain regions responding to 2.5 mg/kg MPD with increased or decreased significant firing rates. The figures on the left-hand side (**A**,**C**,**E**) summarize responses from animals expressing behavioral sensitization, and the right-hand side (**B**,**D**,**F**) summarize responses from those animals expressing behavioral tolerance. The figures in the top row (**A**,**B**) summarize the responses following acute MPD (ED1 MPD/ED1 BL), those in the middle row (**C**,**D**) summarize the changes to the baseline responses (ED10 BL/ED1 BL), and those in the bottom row (**E**,**F**) summarize responses following chronic (rechallenge) MPD (ED10 MPD/ED1 MPD).

A comparison was made between the ratio of neuronal units that responded to acute MPD with increased firing rate (excitation) vs. decreased firing rate (attenuation) among the six different brain regions (Figure 6A), and a significant difference was observed among the six regions (χ^2^ (5, 327) = 53.11; *p* < 0.0001). Further post hoc comparison indicates that the excitation vs. attenuation ratio of neuronal units in response to MPD is significantly lower in the NAc region (adjusted *p* < 0.0001) than the average of all other regions.

### 2.21. Acute Response to 2.5 mg/kg MPD in Animals Expressing Behavioral Tolerance (ED1 MPD/ED1 BL; Table 2C and Figure 6B)

The neuronal recordings following acute 2.5 mg/kg MPD from animals expressing behavioral tolerance to chronic MPD exhibited different proportions in the number of units that responded with a change in firing rate among the six brain regions (χ^2^ (5, 485) = 69.40; *p* < 0.0001). In animals expressing behavioral tolerance, the proportion of neuronal units that responded with a change in firing rate upon acute MPD in the CN (92%) is significantly higher (*p* = 0.03) than the other brain areas, while the proportion of responding neuronal units in region PFC (51%) is significantly lower than the average of all other regions (adjusted *p*-values < 0.0001).

Additionally, the ratios of neuronal units that respond to acute 2.5 mg/kg MPD with increased vs. decreased firing rates are significantly different among these six brain regions (Figure 6; χ^2^ (5, 395) = 19.95; *p* = 0.001). Further post hoc comparison indicates that the ratio of neuronal units with increased vs. decreased firing rates in response to MPD is significantly lower in the NAc (adjusted *p* = 0.016) than the average of all other brain regions.

### 2.22. ED10 BL Neuronal Activity of Animals Expressing Behavioral Sensitization to Chronic 2.5 mg/kg MPD Compared to ED1 BL (ED10 BL/ED1 BL; Table 2B and Figure 6C)

The proportions of units with a change in ED10 BL after six daily 2.5 mg/kg MPD and three washout days are significantly different among the six brain regions (χ^2^ (5, 438) = 247.36; *p* < 0.0001). The proportion of neuronal units with a modified ED10 BL is significantly higher in the CN (99%) when compared to the average response rates of all other regions (adjusted *p*-values 0.02), while the firing rate in region NAc (14%) is significantly lower than the average of all other regions (*p* < 0.0001).

A comparison was also made between the ratio of neuronal units that responded to MPD at baseline with increased vs. decreased firing rates among the six different brain regions (Figure 6C). It was found that the ratios are significantly different among the regions (χ^2^ (5, 346) = 33.49; *p* < 0.0001). Further post hoc comparison indicates that the ratio of neuronal units with increased vs. decreased firing rates is significantly higher in region LC (adjusted *p*-value = 0.017) but significantly lower in region VTA (adjusted *p* = 0.009) than the average of all other regions.

### 2.23. ED10 BL Neuronal Activity of Animals Expressing Behavioral Tolerance to Chronic 2.5 mg/kg MPD Compared to ED1 BL (ED10 BL/ED1 BL; Table 2C and Figure 6D)

The proportions of neuronal units that demonstrated a change in ED10 BL/ED1 BL are significantly different among the six brain regions (χ^2^ (5, 486) = 218.65; *p* < 0.0001). The proportion of ED10 BL neuronal units in region CN (99%) is significantly higher than the average of all other regions (*p* = 0.001). The proportions of neuronal units demonstrating ED10 BL activity that was significantly lower than ED1 BL after six daily MPD treatments and three washout days recorded from the NAc (21%; adjusted *p*-value < 0.0001) and DR (62%; adjusted *p*-value = 0.0002) were significantly different compared to the average response rates of all other regions (*p* < 0.001).

Additionally, the ratios of neuronal units expressing increased vs. decreased firing rates after six daily repetitive MPD treatments are significantly different among the regions (Figure 6D; χ^2^ (5, 377) = 83.53 (*p* < 0.0001). Further post hoc comparison indicates that the ratio of neuronal units expressing increased vs. decreased firing rates is significantly higher in region VTA (adjusted *p* < 0.0001) but lower in region CN (adjusted *p* < 0.0001) than the average of all other regions.

### 2.24. Response to 2.5 mg/kg MPD Rechallenge in Animals Expressing Behavioral Sensitization (ED10 MPD/ED1 MPD; Table 2B and Figure 6E)

The proportions of responding neuronal units to chronic MPD treatment are significantly different among the six brain regions, with χ^2^ (5, 437) = 31.21 (*p* < 0.0001). Specifically, the proportion of neuronal units that responded to MPD rechallenge in region PFC (75%) is significantly lower compared to the average response rates of all other regions (adjusted *p*-values < 0.0001).

An additional comparison was made between the ratios of neuronal units that responded to MPD with increased vs. decreased firing rates to ED10 MPD/ED1 MPD among the six different brain regions (Figure 6E). It was found that the ratios are significantly different among the regions (χ^2^ (5, 387) = 30.30; *p* < 0.0001). Further post hoc comparison indicates the ratio of neuronal units with increased vs. decreased firing rates is significantly higher in CN and PFC when compared to the average of all other regions, with adjusted *p*-values of 0.02 and 0.03, respectively.

### 2.25. Response to 2.5 mg/kg MPD Rechallenge in Animals Expressing Behavioral Tolerance (ED10 MPD/ED1 MPD; Table 2C and Figure 6F)

The proportions of neuronal units that responded to chronic MPD treatment in animals expressing behavioral tolerance to chronic MPD are significantly different among the six brain regions (χ^2^ (5, 486) = 153.8; *p* < 0.0001). The proportion of neuronal units that responded to chronic MPD rechallenge is significantly higher in region CN (99%; adjusted *p*-values = 0.005), while the proportion of responding units in region PFC (33%) is significantly lower than the average of all other regions (adjusted *p*-values < 0.0001).

In addition, it was found that the ratios of neuronal units responding to MPD with increased vs. decreased firing rates are significantly different among these regions (Figure 6F; χ^2^ (5, 397) = 109.90; *p* < 0.0001). Further post hoc comparison indicates the ratios of neuronal units with increased vs. decreased activity in regions CN and NAc are significantly higher than all the other regions (adjusted *p*-value < 0.0001 and 0.002, respectively), while the ratios in regions LC and PFC are significantly lower than the average of all other regions (adjusted *p*-value 0.0001 and 0.004, respectively).

### 2.26. Neuronal Responses to Acute and Chronic MPD Exposure from all Animals (Table 2A)

Table 2A summarizes the effects of MPD on all neuronal units recorded following 2.5 mg/kg MPD. The proportion of responding neuronal units to acute and chronic MPD exposure (ED1 MPD/ED1 BL; ED10 MPD/ED1 MPD), as well as the neuronal activities of ED10 BL after six daily 2.5 mg/kg MPD treatments compared to ED1 BL (ED10 BL/ED1 BL), were significantly different among the six brain regions (χ^2^ (5, 329) = 604.21; *p* < 0.0001). Moreover, this group of animals’ response to 2.5 mg/kg MPD is significantly different (Table 2A) from the neuronal units summarized from the sensitized group (Table 2B; χ^2^ (10, 832) = 711.17; *p* < 0.0001) and the tolerance group (Table 2BC; χ^2^ (11, 445) = 834.26; *p* < 0.0001).

### 2.27. Acute Response to 10.0 mg/kg MPD in Animals Expressing Behavioral Sensitization (ED1 MPD/ED1 BL; Table 3B and Figure 7A)

The proportion of neuronal units that responded to acute treatment of 10.0 mg/kg MPD in the six brain regions of animals expressing behavioral sensitization was compared to the neuronal unit response after chronic 10.0 mg/kg MPD. It was found that the proportions of responding neuronal units are significantly different among the six brain regions (χ^2^ (5, 313) = 18.40; *p* = 0.002). The data were then fit into a logistic regression model, and a post hoc comparison was performed. The proportion of neuronal units that responded acutely to MPD in region LC (77%) is significantly lower compared to the averaged response rates of all other regions (adjusted *p*-value = 0.03).

**Figure 7:**
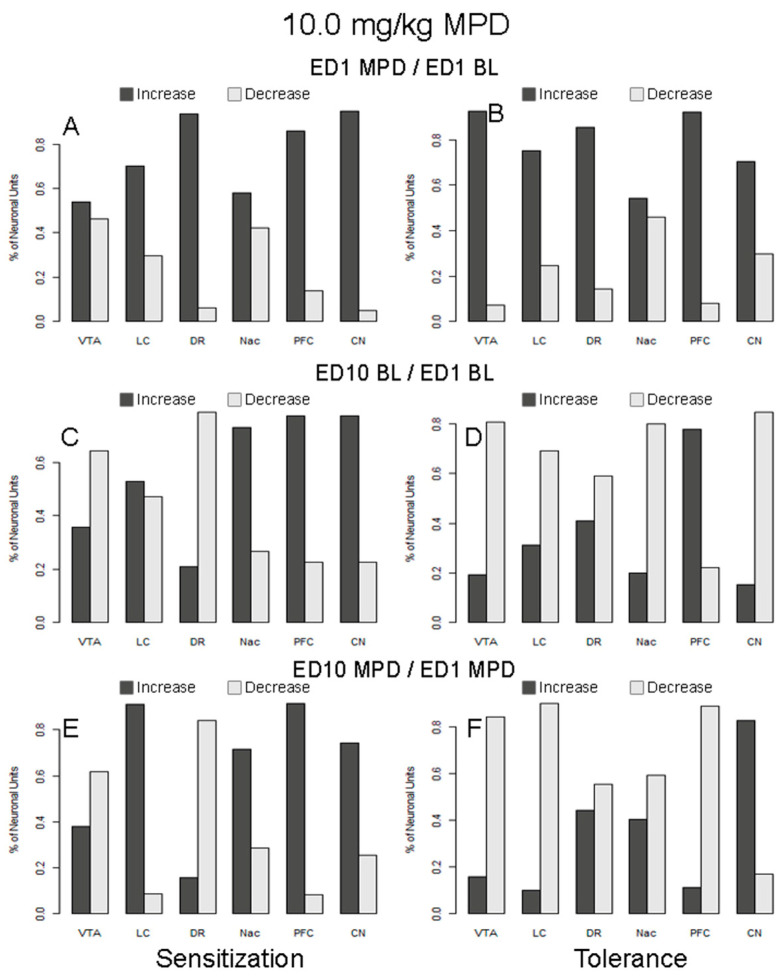
The figure summarizes the percentage of neuronal units from six brain regions responding to 10.0 mg/kg MPD with significantly increased or decreased firing rates. The figures on the left-hand side (**A**,**C**,**E**) summarize responses from animals expressing behavioral sensitization, and the figures on the right-hand side (**B**,**D**,**F**) summarize responses from those animals expressing behavioral tolerance. The figures in the top row (**A**,**B**) summarize the responses following acute MPD (ED1 MPD/ED1 BL), the middle row (**C**,**D**) represent the changes to the baseline responses (ED10 BL/ED1 BL), and the bottom row (**E**,**F**) summarize responses following chronic or rechallenge MPD (ED10 MPD/ED1 MPD).

We also compared the ratio of neuronal units that responded to MPD with increased vs. decreased firing rates in response to MPD exposure among the six different brain regions to a 10.0 mg/kg rechallenge at ED10 (Figure 7A). It was found that the ratios are significantly different among the six brain regions (χ^2^ (5, 280) = 37.66, *p* < 0.0001). Further post hoc comparison indicates that the ratios of neuronal units with increased vs. decreased firing rates following MPD exposure are significantly lower in the regions NAc (adjusted *p* = 0.002) and VTA (adjusted *p*-value = 0.001) than the average of all other regions.

### 2.28. Acute Response to 10.0 mg/kg MPD in Animals Expressing Behavioral Tolerance (ED1 MPD/ED1 BL; Table 3C and Figure 7B)

The neuronal response from animals expressing behavioral tolerance to chronic MPD exhibited different proportions in the number of neuronal unit recordings among the six brain regions (χ^2^ (5, 598) = 26.87; *p* < 0.0001). The proportions of neuronal units responding to MPD acutely in regions PFC (74%) and VTA (72%) are significantly lower than the average of all other regions (Table 3B; adjusted *p*-values 0.007 and 0.008, respectively).

In addition, it was observed that the ratios of neuronal units responding to acute 10.0 mg/kg MPD with increased vs. decreased firing rates are significantly different among these regions (Figure 7B; χ^2^ (5, 507) = 35.64; *p* < 0.0001). Further post hoc comparison indicates that the ratio of neuronal units with increased vs. decreased firing rates is significantly lower in the region NAc (adjusted *p* < 0.0001) than the average of all other regions.

### 2.29. ED10 BL Neuronal Activity of Animals Expressing Behavioral Sensitization to Chronic 10.0 mg/kg MPD Compared to ED1 BL (ED10 BL/ED1 BL; Table 3B and Figure 7C)

The proportions of the neuronal units that changed their ED10 BL after six daily 10.0 mg/kg MPD and three washout days as compared to the initial ED1 BL are significantly different among the six brain regions, with χ^2^ (5, 312) = 167.85 (*p* < 0.0001). The proportion of neuronal units exhibiting modified ED10 BL in NAc (26%) is significantly lower than the average of all other regions (*p* < 0.0001).

We also compared the ratio of neuronal units exhibiting modified BL at ED10 BL/ED1 BL with increased versus decreased firing rates among the six different brain regions (Figure 7C). We found that the ratios are significantly different among these six regions, with χ^2^ (5, 261) = 43.61 (*p* < 0.0001). Further post hoc comparison indicates that the ratio of neuronal units with increased versus decreased firing rates is significantly higher in region PFC (adjusted *p*-value = 0.001) but significantly lower in regions DR (adjusted *p*-value = 0.007) and VTA (adjusted *p* = 0.03) than the average of all other regions.

### 2.30. ED10 BL Neuronal Activity of Animals Expressing Behavioral Tolerance to Chronic 10.0 mg/kg MPD Compared to ED1 BL (ED10 BL/ED1 BL; Table 3C and Figure 7D)

The proportions of the neuronal units that changed their ED10 BL after six daily 10.0 mg/kg MPD and three washout days are significantly different among the six brain regions, with χ^2^ (5, 486) = 218.65 (*p* < 0.0001). The proportion of neuronal units in which the ED10 BL was modified in CN (98%) is significantly higher than the average of all other regions (*p* < 0.0001). The proportion of neuronal units exhibiting the most significantly lower firing rate changes was observed in NAc (6.6%, adjusted *p*-value < 0.0001), compared to the average response rates of all other regions.

In addition, we found that the ratios of neuronal units exhibiting increased versus decreased firing rates in ED10 BL/ED1 BL are significantly different among these regions (Figure 7D; χ^2^ (5, 484) = 61.54; *p* < 0.0001). Further post hoc comparison indicates that the ratio of neuronal units with increased vs. decreased firing rates is significantly higher in the region PFC (adjusted *p* < 0.0001) but lower in the region CN (adjusted *p* = 0.002) than the average of all other regions.

### 2.31. Response to 10.0 mg/kg MPD Rechallenge in Animals Expressing Behavioral Sensitization (ED10 MPD/ED1 MPD; Table 3B and Figure 7E)

The proportions of neuronal units that responded during chronic MPD treatment as compared to the initial 10.0 mg/kg MPD are not significantly different among the six brain regions (χ^2^ (5, 311) = 4.95). The percentages of neuronal units that responded to chronic MPD treatment are above 90% in all six brain regions.

We also compared the ratio of neuronal units that responded to MPD rechallenge with increased vs. decreased firing rates among the six different brain regions (Figure 7E). It was observed that the ratios of neuronal units responding by increasing firing rate vs. decreasing firing rate are significantly different among the six regions (χ^2^ (5, 297) = 82.20; *p* < 0.0001). Further post hoc comparison indicates the ratio of neuronal units that responded with increased vs. decreased firing rates is significantly higher in PFC (adjusted *p*-values < 0.0001) but significantly lower in DR (adjusted *p*-values < 0.0001) when compared to the average of all other regions.

### 2.32. Response to 10.0 mg/kg MPD Rechallenge in Animals Expressing Behavioral Tolerance (ED10 MPD/ED1 MPD; Table 3C and Figure 7F)

The proportions of responding neuronal units to chronic 10.0 mg/kg MPD treatment are significantly different among the six brain regions (χ^2^ (5, 486) = 153.83; *p* < 0.0001). The proportion of neuronal units that responded to chronic MPD rechallenge is significantly higher in region CN (99%; adjusted *p*-values = 0.005), while the proportion of responding neuronal units in region PFC (33%) is significantly lower than the average of all other regions (adjusted *p*-values < 0.0001).

In addition, it was observed that the ratios of neuronal units that responded to 10.0 mg/kg MPD rechallenge with increased vs. decreased firing rates are significantly different among the six brain regions (Figure 7F; χ^2^ (5, 539) = 196.55; *p* < 0.0001). Further post hoc comparison indicates the ratio of neuronal units with increased vs. decreased firing rates in the CN is significantly higher than all the other regions (adjusted *p*-value < 0.0001), while the ratio in region LC is significantly lower than the average of all other regions (adjusted *p*-value = 0.0003).

Table 3A summarizes the effects of MPD on all neuronal units recorded following 10.0 mg/kg of MPD. The proportion of responding neuronal units to acute and chronic MPD exposure (ED1 MPD/ED1 BL; ED10 MPD/ED1 MPD) as well as the neuronal activities of ED10 BL after six daily 2.5 mg/kg MPD treatments compared to ED1 BL (ED10 BL/ED1 BL) were significantly different among the six brain regions (χ^2^ (5, 582) = 732.32; *p* < 0.0001). Moreover, this group of neurons, group “All” (Table 3A—All), is significantly different in response to acute and chronic MPD from the neuronal units summarized in the sensitized group (Table 3B; χ^2^ (13, 053) = 878.55; *p* < 0.0001) and the tolerance group (Table 3C; χ^2^ (14, 572) = 834.26; *p* < 0.0001) to 10.0 mg/kg MPD (*p* < 0.0001).

## 3. Discussion

Methylphenidate (MPD) was first synthesized almost 80 years ago, in 1944, and is now a widely used drug. Knowledge about this drug is still quite vague, as it remains unclear how it specifically affects neuronal and behavioral activities [30,31,32,33,34]. MPD is a central nervous system (CNS) stimulant known for its effectiveness in treating behavioral disorders [35], such as ADHD, and improving cognitive/social function in the elderly. More recently, its unregulated use by ordinary adolescents and adults for cognitive enhancement, concentration improvement, increased productivity, and academic performance has become more popular, as has its abuse for recreational purposes [4,5]. This has become a serious public health concern as the neurological and psychiatric consequences of unrestricted usage of psychostimulants are not widely known [36,37]. Previous investigations by our group have studied the properties of MPD dose–response on the VTA [17], LC [28], DR [28], NAc [17], CN [20], and PFC [20] neuronal activities in adult rats. By recording data from each brain structure separately, it appeared that MPD exerts similar effects in all six above-mentioned brain structures. It is important to verify this empirical conclusion and perform the present study of neuronal recordings from the above six brain areas concomitantly to further elucidate the accuracy of the previous assumptions. Indeed, this study demonstrated that our previous conclusion was wrong.

The present study is unique because it investigates the electrophysiological properties of the VTA, LC, DR, NAc, CN, and PFC simultaneously with animal locomotor behavior, without interference from anesthesia, before and after acute and repetitive (chronic) MPD doses (0.6, 2.5, and 10.0 mg/kg) to investigate the role of each brain area in response to MPD and elucidate what effects acute and/or chronic exposure has on the neuronal population. These six brain areas participate in cognitive function, impulsivity, locomotor activity behaviors, and more [5,38]. The neuronal responses from each of the six brain structures after chronic MPD exposure were also evaluated in terms of increased or decreased firing rate in conjunction with the animals’ behavioral responses or increased/decreased locomotor activity to draw conclusions about neuronal responses obtained from those expressing behavioral sensitization separate from responses obtained from others expressing behavioral tolerance (Table 1, Table 2 and Table 3). Therefore, evaluating the neuronal observations based on the behavioral responses to chronic MPD exposure as compared to the initial (acute) MPD effects provides a unique insight as to whether behavioral sensitization or tolerance was expressed and the concomitant electrophysiological effects.

The main findings of the behavioral observations are that each chronic MPD dose of 0.6, 2.5, or 10.0 mg/kg elicits behavioral sensitization in some animals and behavioral tolerance in others when compared to the initial excitatory effect of the drug (Figure 2). The difference in individual expression for chronic MPD, such as behavioral sensitization or tolerance, was used to identify individual variability in response to MPD. A global transcriptomic analysis of the six brain regions was performed following MPD treatment to examine the obtained transcriptomic profiles with respect to MPD eliciting behavioral sensitization or tolerance in response to the drug, as well as to the effect of MPD eliciting increases or decreases in neuronal firing rates, respectively. This provides a model to identify individual variability in response to MPD.

Indeed, the neuronal response recordings from the VTA, LC, DR, NAc, CN, and PFC obtained from the group expressing behavioral sensitization (Sensitized group) were significantly different from the recordings obtained from those expressing behavioral tolerance to chronic MPD exposure (Tolerance group) as well as from the “All” group. In general, neuronal excitation was observed in the above six brain areas in animals that expressed behavioral sensitization following MPD rechallenge at ED 10 compared to the initial response at ED1, while neuronal attenuation was observed mainly in animals expressing behavioral tolerance as compared to the initial response to acute MPD exposure at ED1. However, each of the six brain areas’ neuronal populations responded differently in terms of the total number of neurons that responded to MPD and the number of responding neurons that demonstrated excitation or attenuation when compared to the initial responses to MPD. The conclusions are as follows: 1. The percentage responsiveness of the DR neuronal units recorded from animals expressing behavioral sensitization exhibits the most responsiveness, or greatest total number of significant responsive units, to acute 0.6 mg/kg MPD (ED1 MPD/ED1 BL) compared to the other five brain areas, and the neurons recorded from the CN exhibited the least responsiveness to acute 0.6 mg/kg MPD. 2. The percentage responsiveness of the LC neuronal units recorded from animals expressing behavioral tolerance to chronic 0.6 mg/kg MPD exhibited the most responsiveness or the greatest total number of significant responsive units, and the neuronal recordings from the NAc and PFC exhibited the lowest percentage of responsive units to the same MPD dose. 3. The DR neuronal activity at ED10 BL after six daily 0.6 mg/kg MPD and three washout days compared to the ED1 neuronal activity at BL (ED10 BL/ED1 BL) from animals expressing behavioral sensitization to chronic 0.6 mg/kg MPD was the most affected, meaning the change in BL activity as a result of chronic 0.6 mg/kg MPD exposure was the greatest. Conversely, the ED10 BL/ED1 BL neuronal recordings from the CN and VTA were least affected by chronic 0.6 mg/kg MPD. 4. The VTA ED10 neuronal BL activities compared to ED1 neuronal BL activity (ED10 BL/ED1 BL) recorded from animals expressing behavioral tolerance to chronic 0.6 mg/kg MPD were the most affected, demonstrating the greatest total number of significant responses, and the PFC neuronal units were the least affected. 5. DR neuronal units exhibited the most responsiveness in the number of significant responsive neuronal units to chronic 0.6 mg/kg MPD as compared to the other brain areas recorded from animals demonstrating behavioral sensitization (ED10 MPD/ED1 MPD), while the VTA neuronal units were the least affected. 6. In the neuronal recordings from the CN in animals expressing behavioral tolerance when comparing ED10 MPD/ED1 MPD, the CN neuronal units exhibited the most significant responsiveness to chronic 0.6 mg/kg, while the PFC neuronal units were the least affected by this MPD dose. 7. In response to acute 2.5 mg/kg MPD (ED1 MPD/ED1 BL), the DR neuronal units exhibited, again, the most significant responsiveness as compared to the other brain areas in behaviorally sensitized animals. 8. In behaviorally tolerant animals, the PFC exhibited the most significant responsiveness to the acute 2.5 mg/kg MPD, while the CN exhibited the least. 9. When comparing the baseline neuronal activity (ED10 BL/ED1 BL) recorded in animals expressing behavioral sensitization to repeated (chronic) 2.5 mg/kg MPD, the neuronal activity recorded from the DR was the most affected in terms of the percentage of neuronal units that responded, and the neuronal activity recorded from the CN was least affected by the six daily 2.5 mg/kg MPD and three washout days. 10. When comparing the baseline neuronal activity at ED10 BL/ED1 BL in animals expressing behavioral tolerance to repeated (chronic) 2.5 mg/kg MPD, the neuronal activity recorded from the LC was the most affected, while that of the CN was least affected by six daily treatments of MPD. 11. When comparing the neuronal response to chronic MPD (ED10 MPD/ED1 MPD) in animals expressing behavioral sensitization, the neuronal units recorded from the DR, LC, NAc, CN, and PFC were equally affected by the 2.5 mg/kg rechallenge at ED10, while the neuronal units recorded from the VTA were the least affected. 12. In each of the six brain areas, chronic MPD elicited excitation in some neurons and attenuation in others when evaluating their firing rates compared to the initial MPD effect. However, the ratio of how many neuronal units responded to MPD by excitation vs. attenuation was significantly different between the six brain areas.

This study revealed that the DR neuronal units recorded from animals expressing behavioral sensitization to chronic 0.6 mg/kg MPD, as compared to the recordings from the other brain areas investigated, exhibited the highest percentage of neuronal units that responded significantly to the 0.6 mg/kg MPD dose by changing their firing rates. However, the LC and CN neuronal units recorded from animals expressing behavioral tolerance to chronic 0.6 mg/kg MPD, as compared to the initial MPD effect, exhibited the highest percentage of neuronal units with a significant response to the smallest MPD dose (0.6 mg/kg). The DR neuronal response recorded following chronic 2.5 mg/kg MPD from animals exhibiting behavioral sensitization exhibited the highest percentage of neuronal units with a significant response to this MPD dose, while the LC, NAc, and PFC neuronal units recorded from animals demonstrating behavioral tolerance exhibited the highest percentage of neuronal units with a significant response by a change in firing rate after chronic 2.5 mg/kg MPD exposure. 

How should this observation be interpreted? We interpreted that in each brain site, there are two dichotomy mechanisms, excitation and inhibition, in response to any stimulus, which is MPD in this case. This excitation-inhibition machinery is essential to how a particular brain area will respond to any stimulus, and the ratio between these two dichotomy responses in each brain structure determines the role of each brain area in response to a given stimulus. Therefore, the different role of each brain area in response to a drug is determined by the ratio of how many neurons in that specific region respond by excitation vs. attenuation. Because different observations were made in each of the six brain areas after the 0.6 and 2.5 mg/kg MPD doses, it suggests that each structure responds in its own unique way to varying MPD doses, as opposed to acting as one large unit with the same responses throughout. Following 10.0 mg/kg MPD, the DR, LC, NAc, CN, and PFC neuronal units responded to MPD in similar ways, suggesting that this high MPD dose of 10.0 mg/kg MPD elicits no specific dose–response effect.

The significant difference observed in the ratios of how many neurons in each brain area respond to MPD by excitation or attenuation suggests that each one of the six brain regions studied exerts a different role in response to MPD. This interpretation is the result of concomitantly recording electrophysiological and behavioral data from all of the above six brain areas and simultaneously evaluating the neuronal responses based on the animal’s behavioral responses to the chronic drug effect as compared to the acute MPD effect, such as sensitization and tolerance.

The behavioral expression of a stimulus is the result of the summation of all the unique neuronal activities in the various brain nuclei participating in a coordinated but not identical response to a particular stimulus. The observation that each brain area responds differently to MPD agrees with the observations using the positron emission tomography (PET) study in humans. These studies report differences in the brain concentrations of DA, NE, and 5HT transporters following MPD exposure [21,39]. Following MPD exposure, 2200 times greater affinity to DAT and 1300 to NET in comparison to SERT were reported [40], while the effect of MPD on neuronal activity firing rate in the DR, VTA, LC, and the other three brain areas does not follow the same levels of the above transporter concentration changes in each area, respectively.

MPD acts primarily as the DA, NE, and 5HT reuptake inhibitor while simultaneously securing the balance between the availability of intra-synaptic DA, NE, and 5HT and the intracellular pool of the neurotransmitters through the interaction with vesicular monoamine transporter 2 (VMAT2) [9]. In monoaminergic neurons, it was observed that MPD affects the redistribution of VMAT2, which is involved in the sequestration of cytoplasmic DA, NE, and 5HT in each brain area differently as an important regulator of neurotransmission [41]. The dopaminergic system is the most well-known for contributing to behavioral expression as well as to addiction behavior. It was reported that administration of MPD led to a 3- to 4-fold increase in both DA and NE in the brain [42]. This increase in DA and NE elevates overall brain activity with several significant behavioral and cognitive effects [43]. There are only a few publications that report that MPD binds to the 5HT transporter and modulates the serotonergic system [5,9,13]. There are limited studies that suggest the serotonergic system and the DR or raphe nuclei, which are the source of 5-HT, mitigate behavior in addiction and craving as well [27,44]. However, this neurophysiological study using neuronal recordings following dose–response experiments on MPD shows that serotonergic signaling from the DR is the most affected following acute and chronic MPD exposure. This study shows that there is no correlation between the monoamine transporter concentration in the brain and the neuronal activity response to MPD. The above observation indicates that each one of the neuronal populations recorded from DR, VTA, LC, NAc, CN, and PFC responds differently to 0.6 and 2.5 mg/kg MPD in animals expressing behavioral sensitization vs. those expressing behavioral tolerance, with no correlation to DA, NE, and 5HT transporter levels. This suggests that each of the above six brain areas has a unique role in the coordinated electrophysiological and behavioral response to MPD. 

In summary, the neuronal composition within each brain region is highly responsive to the neurochemical setting and is regulated by DA, NE, 5-HT, GABA, ACh, and glutamate [45]. MPD treatment increases the DA, NE, and 5-HT levels in each brain area included in this study. DA plays an essential role in regulating the motivation and reward/avoidance behavior, memory, mood, event anticipation, behavioral inhibition, decision-making, and problem-solving of an individual [40,44,46,47,48,49,50]. DA interacts with five different receptors localized in the post-synaptic neurons [45], while NE is important in higher-level cognitive processes such as working memory [51,52] and regulation of attention [53], and this signaling network is activated by MPD. Serotonergic signaling participates in locomotor activities and impulsive behaviors [54], inducing potentially impaired control of impulses and violent behavior when altered [12,55]. Moreover, 5-HT regulates DA activities through its receptors 5-hydroxytrytamine receptor 1B (5-HTR1B) or 5-HTR2A [12,55,56]. All of them participate in response to MPD treatments. In the future, further studies in female SD rat populations should be performed to compare the sex differences of the VTA, LC, DR, NAc, CN, and PFC’s responses to methylphenidate. 

## 4. Material and Methods

### 4.1. Animals

A total of 158 male Sprague-Dawley (SD) rats weighing 170–180 g were obtained from Harlan Indianapolis, IN, USA, and were each placed in a home cage, which also served as the test cage in a controlled room with a 12 h light/dark schedule (light on at 06:00), and given water and food as needed for 5 to 6 days of acclimation prior to surgical implantation of 16 neuronal recording electrodes. A total of 25 animals were used as controls: 12 for time control and 13 for saline control (Figure 1). The experimental group included 45, 41, and 47 animals who received acute and repetitive (chronic) 0.6, 2.5, and 10.0 mg/kg MPD, respectively (Figure 2). 

### 4.2. Animal Preparation

On the day of surgery, the animals were anesthetized with 50 mg/kg pentobarbital. Their heads were shaved, covered with lidocaine hydrochloride topical gel, and placed in a stereotactic head holder. An incision was made on the head to expose the skull by removing the skin, the muscle, and the connective tissue. Bilateral holes were drilled into the skull above the following six brain areas: dorsal raphe (DR), posterior (P) from Bregma 7.8 mm, and lateral (L) from the midline 0.2 mm; locus coeruleus (LC) P-10.4 mm and L-0.4 mm; ventral tegmental area (VTA) P-4.8 mm and L-0.8 mm; NAc-anterior (A) from Bregma 1.7 mm, L-1.5 mm; caudate nucleus (CN) P-0.2 mm, L-3.0 mm; and above the prefrontal cortex (PFC) A-3.2 mm, L-0.4 mm using the Paxinos and Watson [57] Brain Atlas. Additional holes were drilled in the frontal skull for the reference electrodes. At vacant spots in the skull, 6 to 8 screws were inserted laterally to secure the implanted electrodes and the head plug holding the electrodes. Nickel–chromium Teflon-coated electrodes (except at the tips) 60 um in diameter were used as the recording electrodes. Electrodes were inserted individually into the six brain areas bilaterally at initial depths of 6.4 mm, 7.0 mm, 8.4 mm, 6.8 mm, 4.4 mm, and 3.4 mm in the DR, LC, VTA, NAc, CN, and PFC, respectively.

During electrode placement, the neuronal activity was monitored using a Grass P-511 amplifier with its cathode follower connected to an audio monitor and oscilloscope. When spike activity exhibited at least a 3:1 signal-to-noise ratio, the electrodes were fixed to the skull. Otherwise, the electrode was lowered in steps of 5 to 10 um increments until a 3:1 spike-to-noise ratio was obtained, and then the electrodes were fixed to the skull with dental cement. Each electrode was connected to a copper pin, and all the pins were inserted into an Amphenol plug, which was fixed onto the skull with dental acrylic cement. Rats were allowed to recover from the surgical procedure for 5 to 7 days. During this recovery period, rats were placed daily in their home cage in the experimental setup and connected to the wireless head stage (Triangle Bio-System Inc., TBSI, Durham, NC, USA) for about 2 h to adapt and acclimate to the recording system. The first recording day started 5 to 6 days after electrode implantation at post-natal day 60 (P-60) of age, and the animals’ weights were about 220 g. All the experimental procedures were approved by The University of Texas Health Science Center Welfare Animal Committee in Houston, TX, in accordance with the National Institute of Health Guide for Care and Use of Laboratory Animals. All recordings and injections were conducted in the home cages, meaning the home cage was also used as the test cage to eliminate the novelty of the test cage as a potential confounding factor in MPD treatment [14,17,20].

### 4.3. Experimental Procedure (Table 4)

The behavioral locomotor activity and the neuronal activity from the six bilateral brain areas were recorded simultaneously. The open-field computerized animal activity monitoring system (CAAMS, Accuscan Instruments, Inc., Columbus, OH, USA) was used to record behavioral locomotor activity. The CAAMS system consists of 2 arrays of 16 infrared light beams with sensors on the opposite side and spaced every 2.5 cm that cross orthogonally through the plexiglass cage. Because Albino rats cannot see red light, the infrared light beams of the CAAMS system did not interfere with behavioral data collection. The sensor polling frequency was set at 100 Hz. The movement of the rats interrupted the infrared light beams, and each beam reak detected by a sensor was collected as an event by the Accuscan Analyzer and transferred to a computer. Events over a 10 min period were summed, giving six 10 min bins for each hour of recording. These bins were transferred to the OASIS data collection software (Accuscan Instrument Inc., Version 2, Columbus Ohio, USA.), and four indices of behavioral locomotion were compiled for each collection period: total traveling distance (TD)—all forward locomotion in cm; number of movements (NM)—the overall movement in the lower level of the cage counted by the lower arrays; the number of stereotypic movements (NOS)—episodes of repetitive movement in the upper level of the sensors separated by at least 1 s; and the vertical activities (VA) counted by the upper arrays [20].

The Triangular BioSystem Inc. (TBSI, Durham, NC, USA) was used to record the neuronal activities. The head stage contains a 16-channel wireless neuronal recording system consisting of a head stage (weighing less than 4.5 g) and a remote receiver. The TBSI head stage was connected to the rat’s head cap containing the 16 electrode pins and sent electrical signals (sampling notes up to 200 Hz) through a transmitter to a receiver outside the recording cage that was connected to an analog-to-digital converter (Micro 1402-3: Cambridge Electronic Design (CED)). The neuronal activity from each electrode was collected and stored on a PC using the CED Spike 2.7 software. Five groups of animals were used: time control, saline control, and 0.6, 2.5, and 10.0 mg/kg MPD-treated groups. On experimental recording day 1 (ED1), rats were placed in their home cages in a Faraday testing cage to reduce interfering noise. The TBSI wireless head stage was connected to the electrode pins of the skull cap, and animals were allowed to acclimate for an additional 30 min prior to the recording session. After saline injection, behavioral locomotor activity and neuronal activity from all electrodes were recorded simultaneously for one hour post-saline injection. The recording following saline injection serves as the baseline (BL) control activity. Following this 60 min BL recording, additional injections were given of either saline to the control group or 0.6, 2.5, or 10.0 mg/kg MPD to the MPD experimental groups (see Table 4), and recordings (electrical and behavioral) were resumed for an additional 60 min. On ED1, two sets of 60 min were recorded: experimental day 1 BL after saline injection (ED1 BL) and experimental day 1 after acute MPD (ED1 MPD). On ED2 through ED6, rats received daily injections of either saline (the control group) or a single dose of 0.6, 2.5, or 10.0 mg/kg MPD in their home cage to initiate the MPD chronic effect [14,17]. On ED7, 8, and 9, the rats underwent washout days where no injections were given. On ED10, injections and neuronal and behavioral recordings were resumed identically to ED1, i.e., 60 min recordings following saline injection (ED10 BL) followed by 60 min recordings post 0.6, 2.5, or 10.0 mg/kg MPD (ED10 MPD), respectively.

**Table 4:** Summarizes the experimental protocol for the simultaneous behavioral and neuronal recordings from the respective brain regions of all four treatment groups. On experimental recording day 1 (ED1), saline was injected, followed by a 60 min behavioral and electrophysiological recording period, which served as the baseline (BL). The respective treatments (saline, 0.6, 2.5, or 10.0 mg/kg MPD) were then administered, followed by another 60 min recording to obtain the acute effect of the drug. ED2-ED6 consisted of five daily injections of saline or MPD for a total of six consecutive daily injections, including ED1. These daily injections initiated the chronic effect of the drug. ED7-ED9 entails the washout period, during which no injections of any drug were given. Finally, on ED10, saline was once again administered to all animal groups, followed by a 60 min recording period to establish the ED10 BL after six daily injections and three washout days. This was followed by one last injection of saline or respective MPD doses to assess the chronic effects of MPD, which was subsequently compared to the acute MPD effect obtained after ED1. * Indicates the behavioral and neuronal activity recording days.

	Experimental Days (ED)			
Treatment Groups	ED 1 *	ED 2–6	ED 7–9	ED 10 *
Saline	Saline/Saline	Saline	Washout	Saline/Saline
2. 0.6 mg/kg MPD	Saline/0.6 mg/kg MPD	0.6 mg/kg MPD	Washout	Saline/0.6 mg/kg MPD
3. 2.5 mg/kg MPD	Saline/2.5 mg/kg MPD	2.5 mg/kg MPD	Washout	Saline/2.5 mg/kg MPD
4. 10.0 mg/kg MPD	Saline/10.0 mg/kg MPD	10.0 mg/kg MPD	Washout	Saline/10.0 mg/kg MPD

### 4.4. Methylphenidate

Methylphenidate hydrochloride (MPD) was obtained from Mallinckrodt (Hazelwood, MD, USA). MPD was dissolved into a 0.9% isotonic saline solution, and the 0.6, 2.5, and 10.0 mg/kg MPD doses were calculated as a free base. All MPD injections were equalized to a volume of 0.8 mL with 0.9% saline to keep injection volumes equal and were given intra-peritoneally (i.p.). In previous MPD dose–response experiments of the effects of 0.1 to 40.0 mg/kg MPD treatment on rats’ neuronal and behavioral activities, it was observed that neuronal and behavioral activity effects of MPD treatment were obtained from doses of 0.6 mg/kg and above [58]. Patrick et al. [19] reported that regardless of the route of MPD administration, whether it is i.p., intravenous, or oral, the drug concentrations measured in the brain were similar. However, a “one dose fits all” approach does not work as the drug doses differ in terms of efficacy and tolerability profile [30]. Therefore, 0.6, 2.5, and 10.0 mg/kg i.p. MPD doses were selected for this study as low, mid-range, and high doses.

### 4.5. Histological Verification of Electrode Location

At the end of recording on ED10, an overdose of sodium pentobarbital was administered, followed by intracardial perfusion of a 10% formalin solution containing 3% potassium ferrocyanide. A 10 uA DC current was passed through each electrode for 20 s to produce a small lesion at the recording site. The brain was excised and stored in 10% formalin for subsequent histological processing. The position of each of the implanted electrodes was confirmed by the location of the lesion and the Prussian blue spot using the Rat Brain Atlas [57]. Only recordings obtained from electrodes confirmed histologically to be within the aim targets (DR, LC, VTA, NAc, CN, and PFC) that exhibited similar spike amplitude and pattern at ED1 and ED10 following saline and MPD injection were evaluated and presented in this study.

### 4.6. Data Acquisition

#### 4.6.1. Behavioral Data

Each recording day (ED1 and ED10) had two sessions of six 10 min bins: a total of 60 min following saline injection and an additional 60 min following MPD injection. The paired *t*-test was used to determine the effect of acute MPD by comparing the ED1 MPD to ED1 BL, and the chronic effect of MPD was determined by comparing ED10 MPD to ED1 MPD [14,17,20]. Animals that had a significant (*p* < 0.05) increase in locomotor activity at ED10 MPD compared to ED1 MPD (ED10 MPD/ED1 MPD) were classified as exhibiting behavioral sensitization following six daily MPD exposures and three washout days and assigned to the sensitized sub-group.

Animals that exhibited significant decreases (*p* < 0.05) on ED10 MPD/ED1 MPD were classified as exhibiting behavioral tolerance and assigned to the tolerance sub-group. The mean values for these two subgroups, sensitized and tolerant, were exported from OASIS software (Accuscan Instrument Inc., Version 2, Columbus, OH, USA). and each subgroup was further analyzed using Analysis of Variance (ANOVA) with reported measures. In addition, a post hoc Tukey test was run to compare significant (*p* < 0.05) behavioral changes observed between experimental days ED1 and ED10 within each group using SPSS software version 21. Wilcox et al. [59] calculated that six consecutive daily injections are considered chronic as they equal drug exposure for 1.70% of the rat’s lifespan. The human male equivalent of 1.70% lifespan would be 477 days or 15.9 months of treatment, based on an average lifespan of 2.0 years for male SD rats and 78 years for male humans, and thus six repetitive MPD exposures are considered chronic treatment [59].

#### 4.6.2. Neuronal Data

The recorded neuronal activity from each of the electrodes was replayed offline for neuronal spike sorting and statistical analysis using the CED Spike 2.7 software. The neuronal spikes were captured by the program and processed using low-pass and high-pass filters (0.3–3.0 kHz). There were also two window discrimination levels, one for spikes in the positive direction and another for spikes exhibiting a negative direction (Figure 3). The selected spikes that entered the window were used to create a template, which selected 1000 waveform data points from the chosen spikes to be evaluated. The algorithm used to capture the selected neuronal spike pattern allows the extraction of templates that provide high-dimensional reference points that can be used to discriminate consistent and accurate spike sorting regardless of the influence of noise, false threshold crossing, and waveform overlap. All temporally displaced templates are compared with the incoming selected spike events to find the best-fitting template that yields the minimum residue variance. A template matching procedure is then used, so if the distance between the template and waveform exceeds some threshold (80%), the waveforms are rejected to allow spike sorting accuracy in the reconstructed data of about 95%. Spikes with peak durations and patterns outside of these parameters were rejected. The templates that were used to analyze the ED1 file were then loaded onto the ED10 file of the same electrodes from the same animal to evaluate the ED10 neuronal activity. This ensured that the spike amplitude and pattern sorted from ED10 were the same as the ones recorded on ED1. Once spike sorting was completed, the data were exported into a spread sheet. Statistical comparisons were made for each neuronal unit as follows: (1) neuronal unit firing rate after initial MPD exposure (ED1 MPD) was compared to unit firing rates following saline exposure (BL) on ED1 (ED1 BL), i.e., ED1 MPD/ED1 BL; (2) neuronal unit firing rates after saline exposure on ED10 BL was compared with neuronal unit firing rates after saline on ED1 (ED10 BL/ED1 BL) to find out if six daily MPD exposures (ED1 MPD to ED6 MPD of 0.6, 2.5, and/or 10.0 mg/kg MPD) and three washout days modulate the ED10 BL/ED1 BL. Significant differences in ED10 BL/ED1 BL would therefore indicate withdrawal; (3) neuronal unit firing rates after MPD rechallenge at ED10 were compared to unit firing rates after MPD exposure on ED1 (ED10 MPD/ED1 MPD) to determine the MPD chronic effect. Significant changes and the direction of the change (increase or decrease) for each neuronal unit were determined by the critical ratio (CR) test [20]. In addition to the above evaluation of all the recorded units, the data analysis obtained from each of the six brain areas (DR, LC, VTA, NAc, CN, and PFC) was divided into two subgroups based on how the rats responded behaviorally to chronic 0.6, 2.5, and 10.0 mg/kg MDP exposures compared to the initial response to MPD: (1) neuronal activity data recorded from animals expressing behavioral sensitization (ED10 MPD/ED1 MPD) was summed to subgroup “sensitized” and (2) neuronal recordings obtained from animals expressing behavioral tolerance were summed to subgroup “tolerance”.

### 4.7. Statistical Analysis

All statistical tests and analyses are performed in R 4.0.5. The c^2 test was used to examine for significant differences in the percentages of neuronal units responding to MPD among the six brain regions. If the c^2 test result indicates that the percentage of responding neuronal units is significantly different among the six brain regions, with *p* < 0.005, the logistic regression model was then used to fit the data, and post hoc comparisons were performed to identify the regions(s) with significantly different response rates compared to other regions. The post hoc comparisons were made with the function “contrast” in the R 4.0.5 package “emmeans”. The Bonferroni correction method was used to adjust *p*-values for multiple comparisons. The ratio of neuronal units responding to MPD was also analyzed with increased vs. decreased firing rates among six different brain regions using the c^2 test. If there were significant differences among the different brain regions, post hoc comparisons were performed to identify the region(s) with significantly different ratios of neuronal units responding to MPD with increased vs. decreased firing rates when compared to other regions. Sex and gender-based analyses (SGBA) were not performed as the study only included adult male rats.

## 5. Conclusions

In conclusion, this study indicates the importance of recording and observing neuronal and behavioral responses from several brain areas simultaneously, before and following acute and chronic psychostimulant exposure, and evaluating the neuronal recording based on the animal’s behavioral responses to chronic MPD exposure as compared to the initial (acute) effects. In doing so, the different roles of each brain area in response to MPD can be obtained and further elucidated.

## Data Availability

The data supporting the findings of the article are freely available on the University of Texas Health Science Center at Houston research server.
